# Supplementary material for: Cannabimimetic N-Stearoylethanolamine as “Double-Edged Sword” in Anticancer Chemotherapy: Proapoptotic Effect on Tumor Cells and Suppression of Tumor Growth versus Its Bio-Protective Actions in Complex with Polymeric Carrier on General Toxicity of Doxorubicin In Vivo
Source: Pharmaceutics. 2023 Mar 3;15(3):835. doi: 10.3390/pharmaceutics15030835 (PMC10055797; doi:10.3390/pharmaceutics15030835)
Supplement: Supplementary file 1 [file pharmaceutics-15-00835-s001.zip › pharmaceutics-2154401-supplementary.pdf]

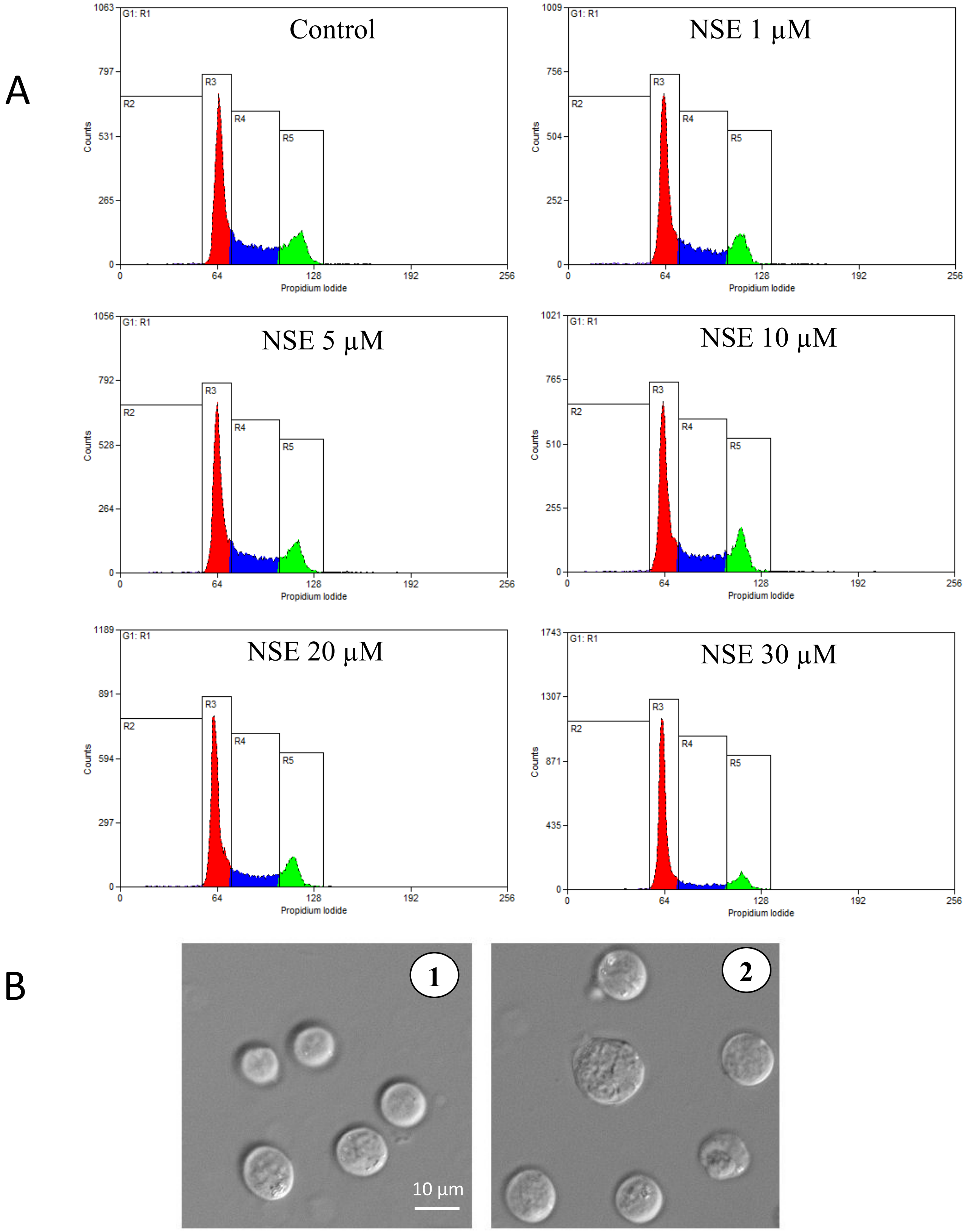

Figure S1. Assessment of cytostatic properties of NSE *in vitro*. (A) Impact of NSE on cell cycle distribution of murine leukemia cells of L1210 line (24 h incubation, PI staining). (B) An increase in diameter of human T-leukemia Jurkat cells after their treatment for 24 h with N-stearoylethanolamine (30 µM). Light microscopy, x40.

Notes: R2 – pre-G1 cells, R3 – G1, R4 – S, R5 – G2

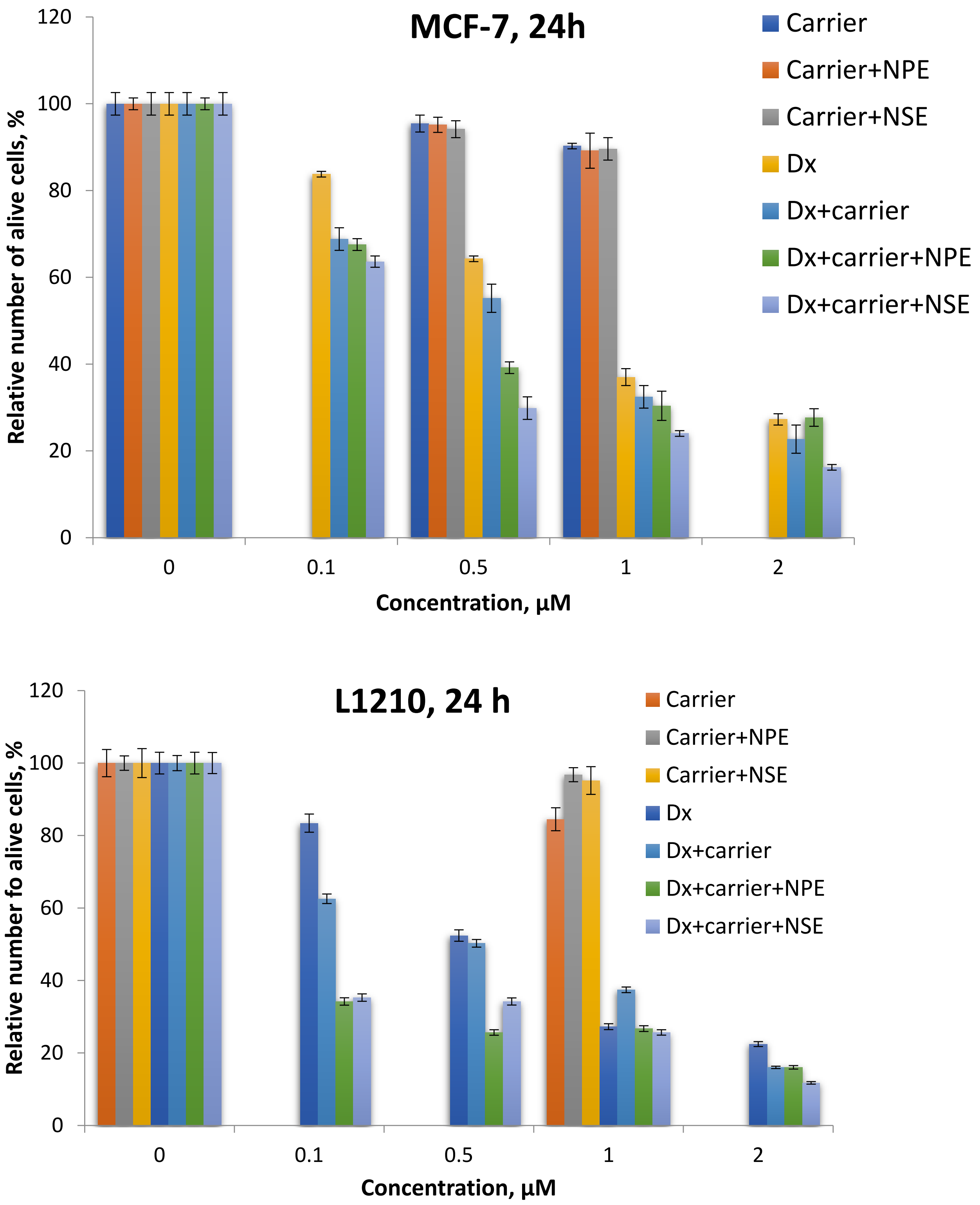

Figure S2. Viability of the indicated cell lines after treatment with Dx, Dx-PC, Dx-PC-NPE and Dx-PC-NSE complexes for 24 h, analyzed by trypan blue exclusion.

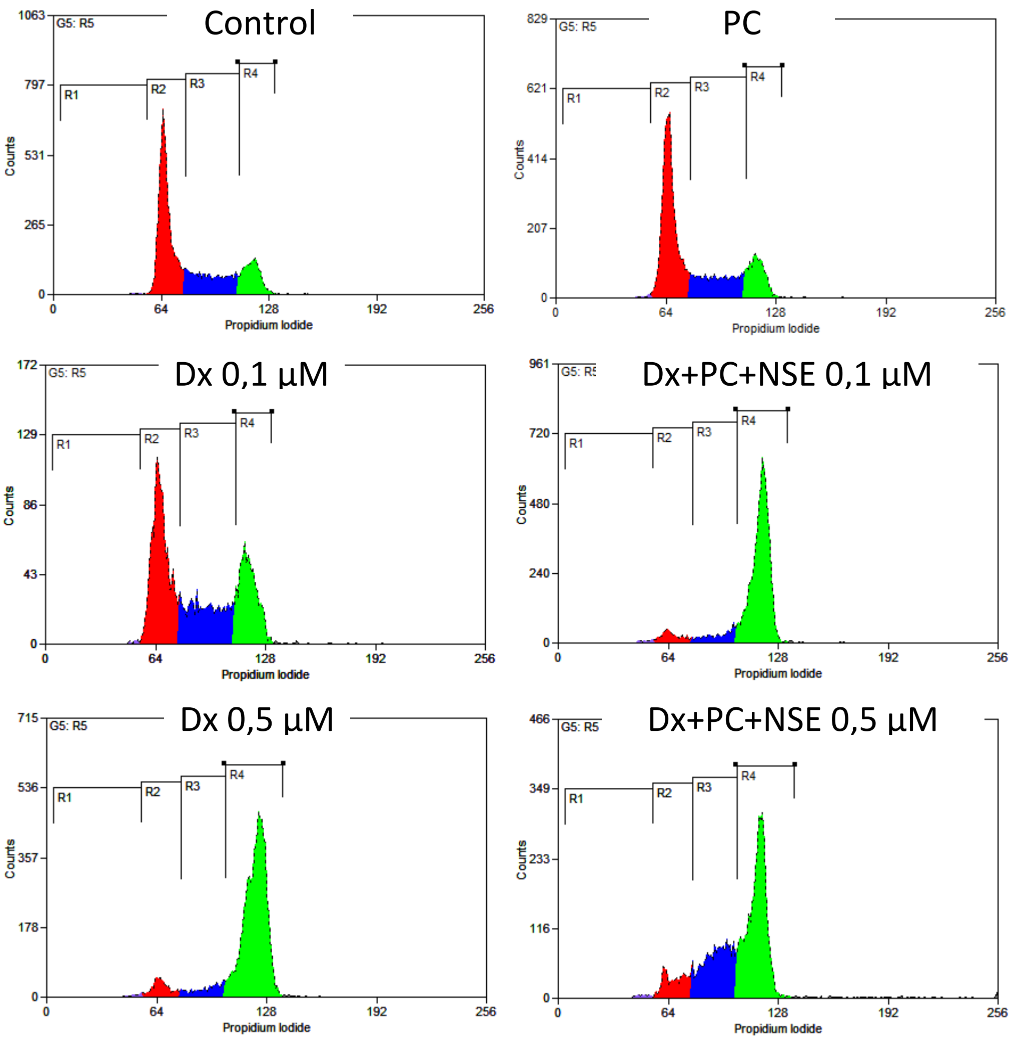

Figure S3. Changes in cell cycle progression in Jurkat cells under treatment with various concentrations of Dx and Dx-PC-NSE, PI staining (24 h incubation).

Notes: R2 – pre-G1 cells, R3 – G1, R4 – S, R5 – G2

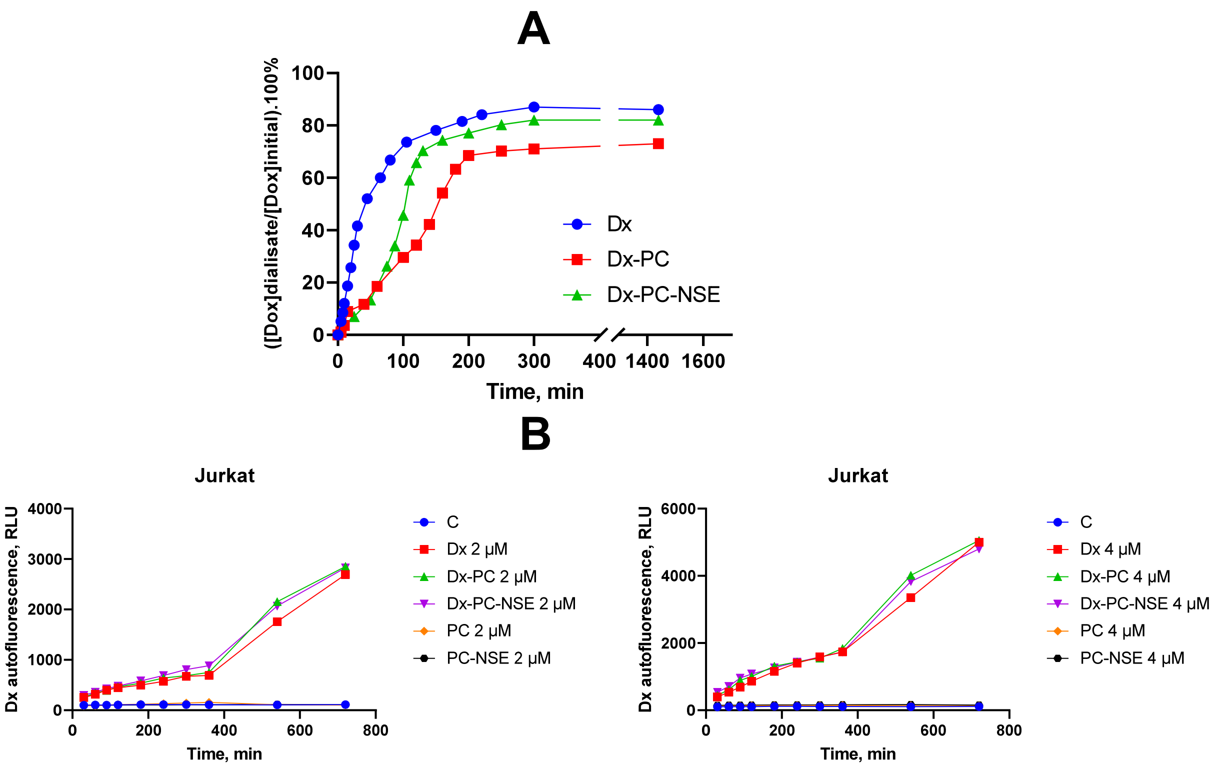

Figure S4. Long-term quantitative analysis of Dx release from its complex with polymeric carrier and its entry into Jurkat T-cells. (A) Time-dependent (0 min – 1440 min) dynamics of a release of free Dx (1) and of Dx immobilized in the micellar complexes Dx-PC (2) and Dx-PC-NSE (3) through the dialysis membrane in aqueous solution (initial concentrations of components loaded into the dialysis tubings were: [NSE] = 0.3 mg/mL; [PC] = 10 mg/mL; [Dx]= 0.3 mg/mL). (B) FACS analysis of time- (30 min-720 min) and concentration-dependent dynamics of Dx, Dx-PC and Dx-PC-NSE entry into Jurkat T-cells *in vitro*.

A

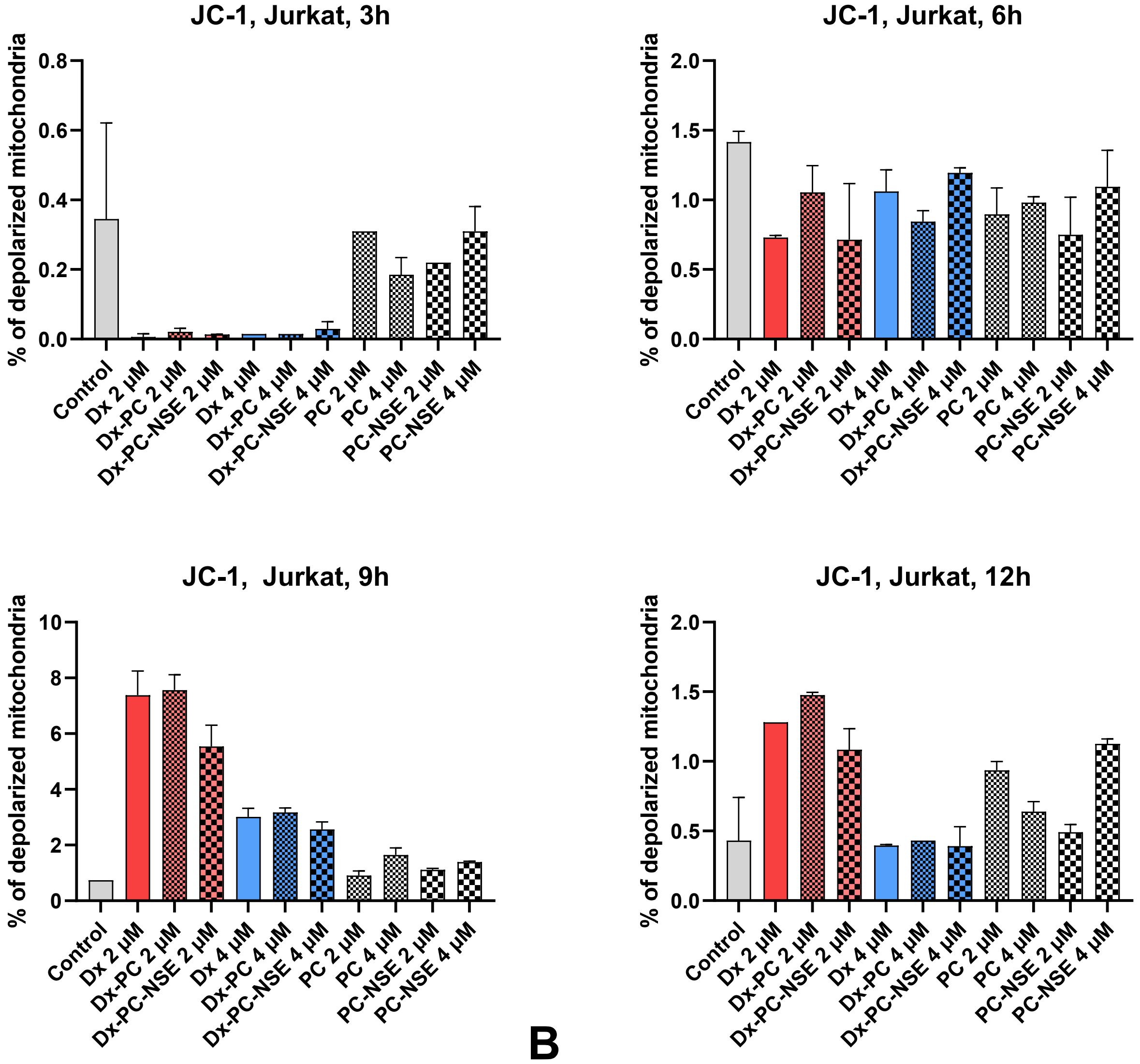

B

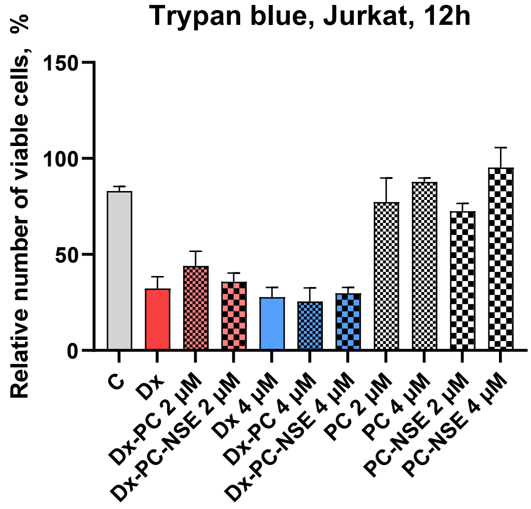

Figure S5. Time-dependent impact of Dx, Dx-PC and Dx-PC-NSE on depolarization of mitochondria in Jurkat cells and their survival. (A) Flow cytometry, JC-1 staining. (B) Trypan blue exclusion assay. One of three experiments delivering comparable data is shown.

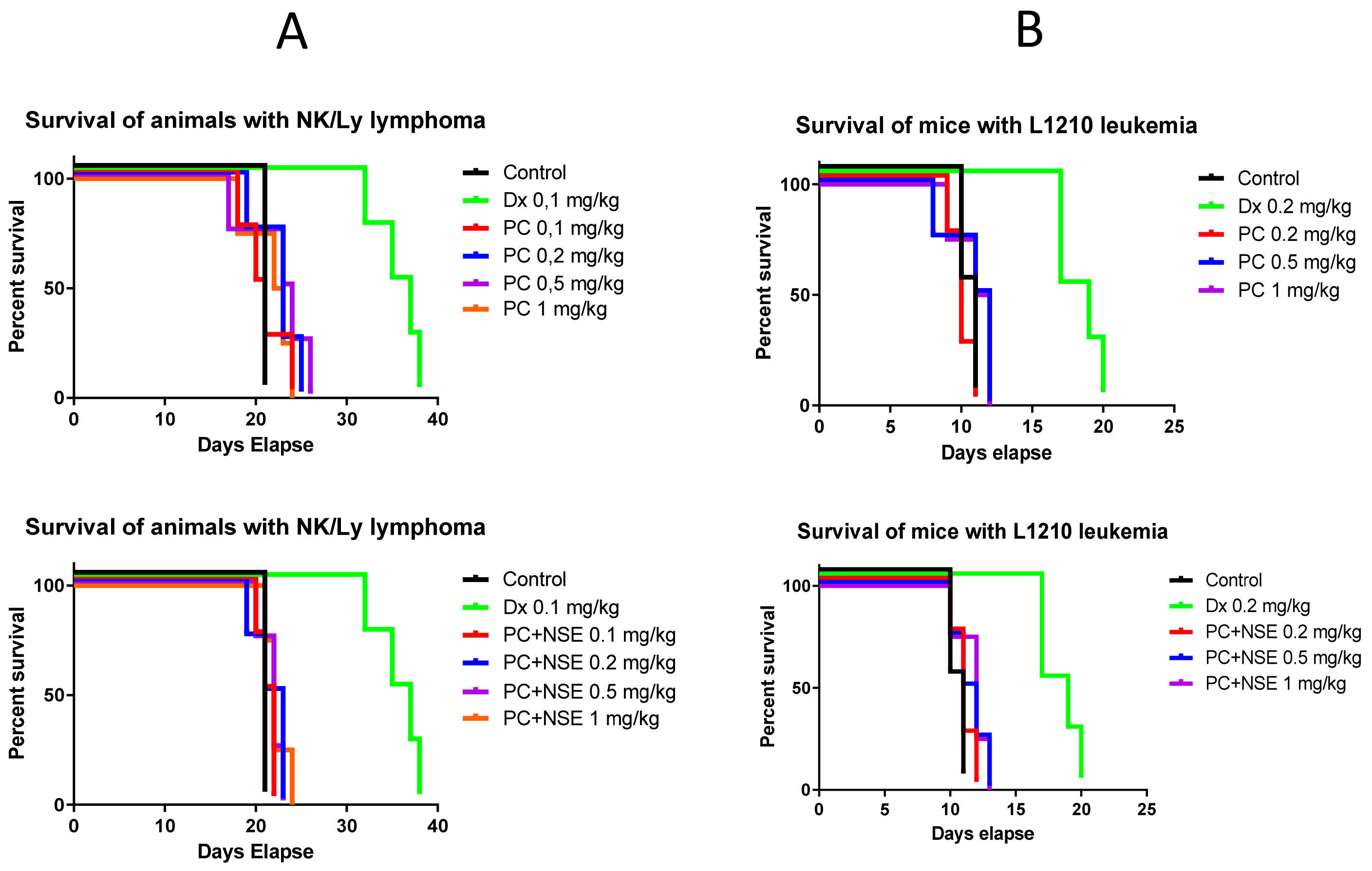

Figure S6. Comparison of therapeutic activity of PC and PC-NSE in various doses (equivalent to those doses of PC and PC-NSE, used to delivery of 0.1 mg/kg-1 mg/kg Dx) towards animals bearing NK/Ly lymphoma (A) and L1210 leukemia (B), respectively.

Table S1. Results of densitometric analysis of Western-blot analysis of time-dependent action of Dx, Dx-PC and Dx-PC-NSE towards human T-leukemia cells of Jurkat line.

Protein	*Lanes*													
	1	2	3	4	5	6	7	8	9	10	11	12	13	14
cl. caspase-3	1,00	1,31	1,57	4,10	12,73	15,46	2,18	3,27	7,28	13,39	3,08	3,07	8,12	11,76
cl. caspase-7	1,00	1,51	1,63	2,79	10,50	14,14	2,21	4,26	6,86	12,01	1,96	1,85	4,71	6,74
DFF45	1,00	1,67	1,79	1,32	1,18	0,51	2,48	2,50	1,30	0,56	1,19	0,86	0,96	0,24
PARP-1	1,00	1,12	1,75	1,25	1,29	0,21	3,02	3,35	0,60	0,31	2,32	1,71	1,12	0,01
cl. PARP-1	0,00	0,00	0,01	0,13	1,88	2,55	0,23	0,36	1,45	3,49	0,12	0,20	1,04	1,71
procaspase-6	1,00	1,85	1,61	1,08	1,57	1,16	1,70	1,82	1,16	1,12	1,14	0,84	0,92	0,65
procaspase-2	1,00	1,46	1,84	1,60	0,64	0,49	2,16	3,24	1,00	0,47	1,57	1,16	0,75	0,11
procaspase-8	1,00	1,45	1,80	1,27	1,31	0,52	1,65	2,21	0,78	0,73	1,18	0,88	0,73	0,13
cl. caspase-8	0,05	0,04	0,04	0,07	1,00	0,39	0,04	0,14	0,24	0,52	0,05	0,06	0,35	0,20
procaspase-9	1,00	1,53	1,57	1,35	1,47	0,96	2,03	2,41	1,49	1,04	1,53	1,13	1,06	0,43
cl.caspase-9	0,14	0,02	0,14	1,00	0,89	0,22	0,01	0,49	0,85	0,27	0,03	0,24	0,41	0,22
procaspase-10	1,00	2,34	2,13	2,24	2,13	1,19	2,56	4,48	2,10	1,05	1,77	0,59	1,18	0,27
FADD	1,00	1,56	2,23	1,84	1,83	1,21	1,74	2,74	0,78	1,04	0,78	0,64	1,02	0,52
AIF	1,00	1,36	1,19	0,92	1,63	1,96	1,37	1,43	1,17	1,60	0,95	0,72	0,85	1,00
Bcl-2	1,00	1,26	0,72	0,70	1,69	2,14	0,65	2,14	0,76	1,73	1,29	0,66	0,50	0,41
Bid	1,00	1,81	2,05	1,90	2,19	1,21	2,11	3,13	1,29	0,50	1,31	0,81	1,14	0,30

Notes:

1 – control
2 – PC+NSE, 2 µM, 24 h
3 – Dx, 2 µM, 3 h
4 – Dx, 2 µM, 6 h
5 – Dx, 2 µM, 9 h
6 – Dx, 2 µM, 12 h
7 – Dx+PC, 2 µM, 3 h
8 – Dx+PC, 2 µM, 6 h
9 – Dx+PC, 2 µM, 9 h
10 – Dx+PC, 2 µM, 12 h
11 – Dx+PC+NSE, 2 µM, 3 h
12 – Dx+PC+NSE, 2 µM, 6 h
13 – Dx+PC+NSE, 2 µM, 9 h
14 – Dx+PC+NSE, 2 µM, 12 h
